# Mapuche Herbal Medicine Inhibits Blood Platelet Aggregation

**DOI:** 10.1155/2012/647620

**Published:** 2011-10-20

**Authors:** Susan Skanderup Falkenberg, Inge Tarnow, Alfonso Guzman, Per Mølgaard, Henrik Toft Simonsen

**Affiliations:** ^1^Department of Plant Biology and Biotechnology, VKR Research Centre Pro-Active Plants, Faculty of Life Sciences, University of Copenhagen, 1871 Copenhagen, Denmark; ^2^Health and Nutrition Division, Chr. Hansen A/S, 2970 Hørsholm, Denmark; ^3^Department of Medicinal Chemistry, Faculty of Pharmaceutical Sciences, University of Copenhagen, 2100 Copenhagen, Denmark

## Abstract

12 plant species traditionally used by the Mapuche people in Chile to treat wounds and inflammations have been evaluated for their direct blood platelet inhibition. Seven of the 12 tested plant species showed platelet inhibitory effect in sheep blood, and four of these were also able to inhibit the ADP- (5.0 *μ*M) and collagen- (2.0 *μ*g/mL) induced aggregations in human blood. These four species in respective extracts (in brackets) were *Blechnum chilense* (MeOH), *Luma apiculata* (H_2_O), *Amomyrtus luma* (DCM : MeOH 1 : 1) and *Cestrum parqui* (DCM : MeOH 1 : 1). The platelet aggregating inhibitory effects of *A. luma* (DCM : MeOH 1 : 1), and *L. apiculata* (H_2_O) were substantial and confirmed by inhibition of platelet surface activation markers.

## 1. Introduction

Chile has an extraordinary variety of plants and animals, thanks to the latitudinal extent of the country and its great altitudinal range. The Chilean Winter Rainfall-Valdivian forest is one of the most exceptional and exposed biodiversity hotspots of the world. It encompasses approximately 40% of Chile's land area and harbours both endemic flora and fauna. About 50% of the 4000 vascular plant taxa found in this area are endemic. Through collaboration, we have access to the traditional medicinal plants from this area [1–3]. The plants examined are traditionally used by the Mapuche people in Chile to treat wounds and associated infections, as shown in Table 1. This paper evaluates the platelet inhibitory capacity of 12 selected plant species. 

Platelet receptors on the surface of the platelets determine the reactivity of platelets and have a wide range of agonists and adhesive proteins [7]. Current antiplatelet therapies target key pathways of platelet activation, including surface receptors and signalling molecules. Aspirin has been the foundation of antiplatelet therapy for over 50 years, and it inhibits platelets by irreversibly acetylating Ser529 of cyclooxygenase 1 (COX1), thereby inhibiting thromboxane A_2_ formation by the platelets. Aspirin has been shown to reduce vascular death in high-risk patients by 15% and nonfatal vascular events by 30%, as evidenced by meta-analysis of over 100 randomized trials [8–10]. Several medicinal plants have direct or indirect antiplatelet effects, many through inhibition of COX1 or 2. Likewise, a variety of fruit extracts have been tested *in vitro* for their antiplatelet property, and tomatoes have been found to have a very high activity [11]. It was showed that tomato extract inhibited both ADP- and collagen induced aggregation by up to 70% but not AA-induced platelet aggregation. Various fruit juices have also been tested, and some flavonoids have been established as inhibitors of collagen-induced platelet activity [12, 13]. The effect of flavonoids is well established, and for coffee, it was showed that the caffeine is not the inhibitor [14] but rather the phenolics that was also found inside the platelets. Many of the effects observed are often due to synergistic effects, which is also seen on tomato and grape juice, and the effect can be expected to be lower for the individual compounds [11–14]. 

The plants collected for this study have been chosen based on their use in the treatment of wounds and inflammatory diseases [1, 2]. Many inflammatory mechanisms are involved in wound healing. Especially, platelets plays a crucial role in haemostasis and thrombosis, and they also play an important role in wound healing, inflammation, antimicrobial host defence, angiogenesis, and tumour growth and metastasis [15]. Therefore, plants used against these or related diseases have been collected. The plants examined in here are collected based on ethnopharmacological data from the Region de Los Lagos in southern Chile, part of the Chilean Winter Rainfall-Valdivian forest [3, 16, 17]. Deforestation threatens this area, and the evaluation of traditional medicine might help to preserve the area with its natural richness. Furthermore, the evaluation also contributes to the preservation of the Mapuche culture, and a sustainable production and/or collection of plants may create an economic foundation as an alternative to the felling of the rainforest.

The aim of the study was a screening of a variety of Mapuche herbal medicine for platelet inhibitory effects. Inhibition on platelet aggregation in sheep blood was chosen as an initial screening method due to the large volumes of blood needed. Plant extracts with activity in sheep blood were subsequently investigated for inhibitory effects on human blood platelets. 

## 2. Material and Methods

### 2.1. Plant Material

The plant species in this study are traditionally used to treat wounds, wound infections, and/or inflammatory ailments by the Mapuche people. The collection have been conducted in February in the years 2001, 2003, 2005, 2006, and 2007 under the supervision of Alfonso Guzman [18]. All plants have been collected in Region de Los Lagos located in Chile's region X. Available plant parts were collected without destroying the population, for example, leaves, stems, flowers, and roots though mainly leaves are used for teas, the preferred preparation in Mapuche traditional medicine (Table 1) [17]. After collection, the plant material was immediately dried at room temperature and transported to Denmark for further studies, where it was kept dry and in darkness until use. Voucher specimens are stored at the Botanical Garden and Museum, University of Copenhagen (C); see Table 1 for voucher specimen number.

### 2.2. Extraction and Sample Preparation

Dried material from 12 different species of Chilean plants was subjected to extraction. 5 mL DCM : MeOH 1 : 1 was added to 0.5 g dry plant material and exposed to ultrasonication for 30 minutes and filtration. This was repeated twice, and the combined extracts were evaporated to dryness. This procedure was repeated using MeOH and finally by H_2_O. The dried extracts were stored at −20°C until used for the aggregation assays. The screening was conducted in each of these three plant extracts, DCM : MeOH 1 : 1, MeOH, and H_2_O. Yield of extractions are given in Table 2.

### 2.3. Preparation of Samples for Aggregation and Flow Cytometry Assays

The dried extracts were dissolved in DMSO : EtOH 1 : 4 in order to reach a concentration of 20 mg/mL, only extracts that was fully redissolved where taken forward. Tested extracts are listed in Table 2. The DMSO : EtOH samples were diluted in sterile filtered HEPES-tyrode's buffer pH 7.4 (137 mM NaCl, 2.7 mM KCl, 1.0 mM MgCl_2_, 12 mM NaHCO_3_, 0.4 mM Na_2_HPO_4_, 5.5 mM glucose, 10 mM HEPES) for aggregation assays, and in HEPES-tyrode's buffer with 0.5% BSA for flow cytometry assays to a final concentration of 1 mg/mL. The DMSO : EtOH samples diluted in HEPES-tyrode's buffer was added to PRP as a 1 : 10 dilution. HEPES-tyrode's buffer containing 0.5% DMSO : EtOH 1 : 4 was used as vehicle control. Sample and vehicle control were incubated in PRP at 37°C for 30 min before aggregation experiments. Final concentration of plant extract and vehicle control in the aggregometer was 0.1 mg/mL. A pure MeOH aliquot was treated as an extract, and the effect of DMSO and MeOH was observed in all assays but did not show any significant effect.

### 2.4. Platelet Aggregation Assay

Platelet aggregation assays were performed in sheep and human blood in duplicates. Sheep blood was used for the bulk screenings, where a large volume of blood was needed. Human blood from the authors SSF (female, 32) and IT (female, 37) was used for selected extracts to verify an inhibitory effect seen in sheep blood at first screening. None of the human volunteers had been exposed to antiplatelet medication for at least 2 weeks prior to blood sampling. Venous blood was drawn with minimal stasis using a 21 G needle into Vacutainer tubes containing 3.2% (0.129 M) sodium citrate (1 : 9), after discarding the first 2 mL.

Platelet-rich plasma (PRP) was prepared by centrifugation of citrated blood at 150 ×g for 10 minutes (human blood) or 1200 ×g for 3 minutes (sheep blood) at 21°C. Autologous platelet-poor plasma (PPP) was prepared by centrifugation of the remaining blood at 3000 ×g for 10 min at 21°C. In each sample, the platelet count of the PRP was determined by an automated counter (Medonic CA 620Vet, Boule Medical AB, London, UK), and based on the count PRP was adjusted to the standard concentration (250,000 platelets/*μ*L). Platelet aggregation was performed by standard procedures (Chronolog VS700, Chronolog Corp., Haverton, Pa, USA) with the following modifications: 225 *μ*L PRP and 25 *μ*L agonist were used in all experiments. Platelet poor plasma with DMSO : EtOH samples diluted 1 : 20 in HEPES-tyrode's buffer was used as reference to eliminate bias of the extract colouring in the test. Final concentrations of the agonists in sheep samples were 5 *μ*g/mL collagen (Chronolog Corp, Haverton, Pa, USA) and 5 *μ*M adenosine diphosphate (ADP—Bio/Data, Horsham, PA) and 2 *μ*g/mL collagen and 5 *μ*M ADP in human samples. Extract and vehicle control samples were analysed in parallel.

Aggregation response was recorded using the Aggrolink software (Chronolog Corp). Maximal aggregation (MA) was recorded in order to obtain a % inhibition of plant extract, comparing the vehicle control (HEPES-tyrode's buffer including BSA) with that of the plant extract %inhibition = (MA vehicle control − MA extract)/MA vehicle control ∗ 100%. 

The experiments were approved by the Animal Experiments Inspectorate under the Danish Ministry of Justice. All human blood used was drawn from the authors themselves.

### 2.5. Initial Experiments in Aggregometer

Arachidonic acid (AA), ADP and collagen were tested in several concentrations in sheep blood in order to find the most suitable agonists and the appropriate concentrations of these. 

Based on the results from the initial experiment it was decided to test AA in 500 *μ*M, ADP in 5.0 *μ*M, and collagen in 2.5 *μ*g/mL in an initial experiment, and from that, it was decided to use ADP and collagen to all future experiments in the aggregometer. The tested ADP concentration at 5.0 *μ*M was suitable, whereas the collagen concentration was increased from 2.5 *μ*g/mL to 5.0 *μ*g/mL. The aggregation percentage in the collagen-induced reaction with vehicle control was 61%, and by increasing the concentration of agonist, the aggregation percentage would hopefully also increase in order to get closer to the desired 70%. AA was not used in any further experiments. 

ADP and collagen were used as agonists based on the initial experiments. For human blood, it was decided to lower the collagen concentration to 2.0 *μ*g/mL due to the observed aggregation. The ADP concentration was the same (5.0 *μ*M) as in the experiment with sheep blood.

To establish the plant extract testing concentration, the MeOH extract of the pharmacologically well described of *Drimys winteri* [6] was tested in three different concentrations, 10, 1, and 0.1 mg/mL. All three gave high inhibition with 0.1 mg/mL yielding 38% (ADP agonist) and 90% (collagen agonist) inhibition of sheep blood aggregation. Taking into account that the 0.1 mg/mL was also significantly easier to dissolve in the testing buffers, it was decided to use this concentration throughout the screening. This would still give positive results for potent aggregation inhibitors.

### 2.6. Flow Cytometry

The DMSO : EtOH samples from *Amomyrtis luma *and* Luma apiculata* (1 mg/mL) diluted in HEPES-tyrode's buffer containing BSA pH 7.4 was used for flow cytometry experiments. 0.5% EtOH in HEPES-tyrode's buffer with BSA was used as vehicle control. Citrated human blood was incubated with DMSO : EtOH/HEPES-tyrode's samples (final concentration of 0.1 mg/mL) or vehicle control at 37°C in 30 minutes.

Samples were assayed within 15 minutes from venipuncture. Microcentrifuge tubes were prepared containing a mixture of either HEPES-Tyrode's buffer, phycoerythrin (PE) conjugated anti-CD62P (Santa Cruz Biotechnology, Santa Cruz, Calif, USA), fluorescein isothiocyanate (FITC) conjugated PAC-1 (Becton Dickinson, San Jose, Calif, USA), or HEPES-Tyrode's buffer, PE-Cy5-conjugated anti-CD42b (Becton Dickinson), fluorescein isothiocyanate (FITC) conjugated PAC-1 (Becton Dickinson, San Jose, Calif, USA) and eptifibatide. To both mixes platelet agonist was added for the detection of platelet surface P-selectin and activated GPIIb/IIIa. Pilot experiments using several different agonist concentrations were performed to identify agonist concentrations giving maximal and submaximal platelet activation. Final concentrations of agonists in the reaction mixture were 1 or 5 *μ*M of thrombin receptor activating peptide (TRAP, Sigma-Aldrich, Brondby, Denmark), 0.5 or 20 *μ*M of ADP (Bio/Data Co., Horsham, Pa, USA), or no agonist (HEPES-Tyrode's buffer). All ADP and TRAP dilutions were made as batches and stored at −20°C along with vehicle control for the controls to minimize dilution variation. Antibody mixtures were prepared as batches and kept at 4°C. After incubation, P-selectin and activated GPIIbIIIa samples were fixed by 1% formaldehyde in HEPES-saline. Samples were analyzed in an FACSCalibur (Becton Dickinson) flow cytometer. Platelets were identified by light scatter properties and expression of CD42b. All samples were tested in triplicates.

## 3. Results

### 3.1. Platelet Aggregation in Sheep Blood

After conducting the initial experiments, a total of 33 extracts, from 12 different plants were screened in ADP (5.0 *μ*M) and collagen (5.0 *μ*g/mL) induced aggregations in the aggregometer. Table 2 shows the average reading of duplicates and whether or not the plant extracts were able to inhibit ADP and collagen induced aggregation. All extract was compared towards the vehicle control and if the observed aggregation was 20% lower for extract test than for the vehicle is was concluded that the extract inhibited aggregation. Additionally, all extracts was tested with two different inducers, this further support the validity of the inhibition results. 

Plant samples that in a convincing way were able to inhibit the aggregation in sheep blood were subsequently tested in a similar experiment with human blood. The below seven plants in the respective extracts were the ones chosen to be tested again.

The DCM : MeOH 1 : 1 extracts of *Amomyrtus luma, Blechnum chilense*, *Cestrum parqui, Lomatia ferruginea*, and *Pseudopanax laetevirens* were active as were the MeOH extracts of *A. luma*, *Anthoxanthum utriculatum B. chilense, C. parqui*,* Luma apiculata, *and* P. laetevirens*, and the water extracts of *A. luma*, *C. parqui, *and *L. apiculata*. These samples were all chosen to be tested in human blood.

### 3.2. Platelet Aggregation in Human Blood

Seven of the species tested in sheep blood was tested in human blood. The activity is listed in Table 3. The four plants *A. luma* (DCM : MeOH 1 : 1 extract), *B. chilense* (MeOH extract), *C. parqui* (DCM : MeOH 1 : 1 extract), and *L. apiculata* (H_2_O extract) showed inhibitory effect in both sheep and human blood in both ADP- and collagen induced aggregations (see Tables 2 and 3). Furthermore, the H_2_O extract from *A. luma* showed inhibition in the collagen induced aggregation.

### 3.3. Flow Cytometry

In order to confirm the obtained results from the aggregation experiments the *A. luma* DCM : MeOH 1 : 1 extract and the *L. apiculata *H_2_O extract were tested for inhibition of platelet surface activation markers flow cytometry. 

Table 4 shows the tested extracts and the percent inhibition of PAC1 MFI and CD62P (P-selectin) MFI by addition of ADP (0.5 *μ*M and 20 *μ*M) and the human specific inducer TRAP (1.0 *μ*M and 5.0 *μ*M). PAC1 and CD62P are both markers of platelet activation, and in order to be assigned an inhibitory effect the extracts should inhibit both activation markers using both agonists at all concentrations.

The extracts from *L. apiculata* and *A. luma* showed clearly inhibitory effect of both PAC1 MFI and CD62P MFI in the tested ADP concentrations as well as with the addition of 1.0 *μ*M TRAP, whereas only a slight inhibitory effect is observed when 5.0 *μ*M TRAP was added. TRAP was used in the flow cytometry assays since it is a human specific platelet inducer.

## 4. Discussion

The four plants *Amomyrtus luma*, *Blechnum chilense*, *Cestrum parqui*, and *Luma apiculata* showed inhibitory effect in both sheep and human blood in both ADP and collagen induced aggregations. Of these *L. apiculata* (H_2_O extract) and *A. luma* (DCM : MeOH 1 : 1 extract) was the most prominent candidates for further examinations. The two extracts were examined using platelet specific markers PAC1 and CD62P and the human-specific inducer TRAP and ADP in a flow cytometry assay. PAC1 and CD62P (P-selectin) does not bind to resting platelets but only to activated platelets [19]. These studies showed clear platelet inhibitory effect on platelet surface activation markers by the two markers as shown in Table 4. The effect observed in the flow cytometry confirms the results seen in the aggregometer.

The ethanol extract of the leaves of *A. luma* has been shown to contain 1-phenylpentan-3-one (4.6/8.5%) and 1-phenylhexan-3-one (3.5/12.3%) as well as *β*-caryophyllene oxide (10.7/6.6%) and linalool (59.3/11.3%) [20], of these the *β*-caryophyllene oxide has been shown to spontaneously aggregate blood platelets [21] at 100 *μ*g/mL concentrations. This effect contradicts the observed effect of the extract, where aggregations was inhibited and suggest a strong inhibition of the organic extracts of *A. luma* since *β*-caryophyllene oxide would have been extracted with both DCM : MeOH 1 : 1 and to some extend also MeOH. The presence of *β*-caryophyllene oxide could be part of the explanation on why no inhibition was observed for the MeOH extract using ADP as an inducer. Further studies are needed to determine the active constituents in *A. luma*. 

The MeoH extracts of *B. chilensis *have previously been shown to have antimicrobial effects [3].* L. apiculata* have previously been shown to have xanthine oxidase inhibitory activity (30% inhibition at 50 *μ*g/mL EtOH : H_2_O 7 : 3 extract) [22], and antiviral activity on herpes (IC_50_ = 100 *μ*g/mL, EtOH extract) [23]. But none of these studies provides information to what could be active constituent, and no phytochemical data was found for these two species. COX inhibitory activity indirectly inhibiting P-selectin expression on human platelets [24]. It has been demonstrated that caffedymine from cocoa, have COX inhibitory activity, with 43% inhibition of COX-1 at 0.01 *μ*M, and that caffedymine suppress P-selectin (CD62P) expression on platelets by 33% at a concentration of 0.05 *μ*M [24]. The inhibition of COX enzymes may be a main contributing factor to suppressing P-selectin expression [24], which could be the effect observed with extracts of *L. apiculata* and *A. luma*. In order to confirm or invalidate this theory, COX inhibitory effect of *L. apiculata* and *A. luma* would have to be examined. Several plant extracts have already been tested for their COX activity [25] and this would need to evaluated along with determination of the active constituents.

It has previously been shown, that a MeOH : H_2_O 1 : 1 extract from *C. parqui* was able to inhibit aggregation of human blood platelets induced by ADP [26]. This confirms that some extracts from *C. parqui* are able to inhibit ADP induced platelet aggregations. However, the same was not observed in an AA-induced platelet aggregation, which implies that, the extracts anti-inflammatory activity did not implicate the inhibition of the cyclooxygenase pathway, that has been seen in other studies [27]. A suggestion is that the extract inhibited a site upstream of AA metabolism, since the case might be that ADP has triggered the release of AA in the pathway [26]. These data could explain the data observed and the two datasets suggest that *C. parqui* contains several active constituents. The plant itself have long been known to cause poisoning in cows, and it has been shown that the toxicity is in the organic phase that contained low molecular weight phenols [28], among these flavonoids that as mentioned have been shown to have antiplatelet activity. With more than 150 publication on *C. parqui* and its pharmacology and several toxic and pharmacologically active terpenoids isolated from the plant [29] further studies are not of high priority.

## 5. Conclusion

In the present work, four Chilean plant species were shown to inhibit platelet aggregating induced by ADP and collagen in both sheep and human blood. The four species were *Blechnum chilense* (MeOH extract), *Luma apiculata * (H_2_O extract), *Amomyrtus luma* (DCM : MeOH 1 : 1 extract) and *Cestrum parqui* (DCM : MeOH 1 : 1 extract). The platelet aggregating inhibitory effects of *A. luma* (DCM : MeOH 1 : 1 extract) and *L. apiculata* (H_2_O extract) were furthermore confirmed by inhibition of platelet surface activation markers. 

At present, there is still a great need for preventative and therapeutic, anticoagulant medicine, and plants and their fruits seem to constitute possible alternatives to drugs currently used. It is of great interest to explore this inhibition further for the three species *B. chilense, L. apiculata, A. luma*. 

## Figures and Tables

**Table 1:** Overview of the plants examined for blood aggregation inhibition, including voucher number, Latin name, local name, and use. All the plants have been collected in region X in the Valdivian Coastal Range Forest. L: leaf, S: stem, R: root, W: whole plant, T: thorn, and F: flower.

Family	Voucher number	Latin plant name	Collected part	Common name	Local use
Araliaceae	PM01-44	*Pseudopanax laetevirens* (Gay.) Baill.	L, S	Sauco	Leaves, fruit and bark are used for wound healing, as anti-inflammatory, laxative and as diuretic [3]
Asteraceae	PM01-28	*Baccharis absinthioides *Hook. & Arn.	L		*Baccharis *leaves are used for wound healing, as anti pyretic and analgesic [4]
Blechnaceae	PM01-18	*Blechnum chilense *(Kaulf.) Mett.	L, S, R	Costilla de vaca	The whole plant is used towards gonorrhoea and wound and eye infections [3]
Gunneraceae	PM01-09	*Gunnera chilensis *Lam.	L, S	Nalca	Stem and root are used against uterus pains, as haemostatic and anti-inflammatorial [3]
Lamiaceae	PM06-38	*Satureja multiflora *Briq. in Engl & Prantl	L	Oreganillo	Leaves used for digestive problems [5]
Malvaceae	PM01-10	*Corynabutilon vitifolium* (Cav.) Kearney	L, S	Huella	Bark, stem and leaves are used for liver diseases and uterus contractions [3]
Myrtaceae	PM03-24	*Amomyrtus luma *(Molina) D. Legrand & Kausel	L, S	Luma	Leaves are used to decrease blood pressure and cholesterol levels, and to treat liver diseases [3]
Myrtaceae	PM01-40	*Luma apiculata *(DC.) Burret	L, S	Arrayán, Quetri	Leaves are used to treat diarrhea, dysentery, ingestion [6]
Myrtaceae	PM01-16	*Ugni molinae*	L, S	Murta	The fruit is stimulating and refreshing [3]
Onagraceae	PM+1-19	*Fuchsia magellanica *Lam.	L, S		Leaves are used as antipyretic, blood pressure regulator, diuretic and wound healing [3]
Poaceae	PM03-32	*Anthoxanthum utriculatum *(Ruiz & Pav.) Y. Schouten & Veldkamp	L	Ratonera	Roots are used traditionally [3]
Proteaceae	PM03-25	*Lomatia ferruginea *(Cav.) R.Br.	L, R		*Lomatia *leaves and bark are used as laxative, expectorant and as anti-inflammatory [3]
Solanaceae	PM05-35	*Cestrum parqui *L'Hér	L	Palqui	Leaves are used to relief fevers, and towards skin diseases [3]
Winteraceae	PM07-05	*Drimys winteri *J.R. & G. Forster	L, B		Leaves are used as antipyretic, in wound healing, as diurectic anti inflammatory agent, and against ulcers [3]

**Table 2:** Mapuche medicinal plant extracts tested in sheep blood. The agonists are ADP (5.0 *μ*M) and collagen (5.0 *μ*g/mL) and the obtained aggregations are shown in percentage. Extracts are tested in 0.1 mg/mL end concentrations. The aggregation is shown for both extract and vehicle control. If the extract aggregation is 20% lower than that of the vehicle control inhibition is observed.

Plant	Extract	Extract Yield (% dw)	Agonist	% Aggregation (extract)	% Aggregation (vehicle control)	% Inhibition	Inhibition
*Amomyrtus luma* (leaf)	DCM : MeOH 1 : 1	6.8	ADP	47	88	47	Yes
			Collagen	25	85	71	Yes
	MeOH	14.4	ADP	61	60	−2	No
			Collagen	30	83	64	Yes
	H_2_O	6.7	ADP	45	68	34	Yes
			Collagen	23	86	73	Yes

*Anthoxanthum utriculatum* (leaf)	DCM : MeOH 1 : 1	5.2	ADP	38	49	22	Yes
			Collagen	106	124	15	*1
	MeOH	4.9	ADP	33	68	51	Yes
			Collagen	22	77	71	Yes
	H_2_O	4.8	ADP	50	51	2	No
			Collagen	39	75	48	Yes

*Blechnum chilense* (leaf)	DCM : MeOH 1 : 1	2.8	ADP	52	62	16	No
			Collagen	26	44	41	Yes
	MeOH	1.8	ADP	38	52	27	Yes
			Collagen	1	18	94	Yes

*Cestrum parqui* (leaf)	DCM : MeOH 1 : 1	7.6	ADP	49	81	40	Yes
			Collagen	27	91	70	Yes
	MeOH	3.7	ADP	44	77	43	Yes
			Collagen	30	83	64	Yes
	H_2_O	15.9	ADP	73	82	11	*1
			Collagen	52	82	37	Yes

*Corynabutilon vitifolium* (leaf)	DCM : MeOH 1 : 1	3.6	ADP	48	54	11	No
			Collagen	44	35	−26	No
	MeOH	3.7	ADP	39	59	34	*1
			Collagen	48	49	2	No

*Fuchsia magellanica* (leaf)	DCM : MeOH 1 : 1	12.2	ADP	45	45	0	No
			Collagen	10	12	17	No
	MeOH	6.2	ADP	30	52	42	Yes
			Collagen	32	38	16	No

*Gunnera chilensis* (leaf + stem)	DCM : MeOH 1 : 1	13.1	ADP	50	59	15	No
			Collagen	49	64	23	Yes
	MeOH	3.3	ADP	40	56	29	*1
			Collagen	35	65	46	Yes
	H_2_O	6.5	ADP	50	71	30	*1
			Collagen	68	56	−21	*1

*Lomatia ferruginea* (leaf)	DCM : MeOH 1 : 1	2.7	ADP	24	55	56	Yes
			Collagen	9	33	73	Yes
	MeOH	1.1	ADP	43	50	14	No
			Collagen	6	55	89	*2
	H_2_O	6.9	ADP	57	43	−33	No
			Collagen	30	50	40	*1

*Luma apiculata* (leaf)	DCM : MeOH 1 : 1	4.5	ADP	51	65	22	*1
			Collagen	42	75	44	*1
	MeOH	4.9	ADP	18	52	65	Yes
			Collagen	20	64	69	Yes
	H_2_O	9.1	ADP	19	56	66	Yes
			Collagen	21	74	72	Yes

*Pluchea absinthioides* (leaf)	DCM : MeOH 1 : 1	8.9	ADP	47	57	18	*1
			Collagen	17	39	56	Yes
	MeOH	2.9	ADP	48	50	4	No
			Collagen	10	36	72	Yes
	H_2_O	4.4	ADP	43	51	16	No
			Collagen	9	51	82	*2

*Pseudopanax laetevirens* (leaf)	DCM : MeOH 1 : 1	7.3	ADP	45	59	24	Yes
			Collagen	23	61	62	Yes
	MeOH	6.2	ADP	43	57	25	*1
			Collagen	4	60	93	Yes
	H_2_O	13.3	ADP	50	66	24	Yes
			Collagen	8	6	−33	No

*Satureja multiflora * (leaf + stem)	DCM : MeOH 1 : 1	7.2	ADP	44	74	41	Yes
			Collagen	31	81	62	*1
	MeOH	7.1	ADP	60	67	10	No
			Collagen	44	125	65	*1
	H_2_O	14.3	ADP	61	64	5	No
			Collagen	11	43	74	*2

*1:  Aggregation curve and output % does not correlate, and the result is doubtful.

*2:  Aggregation curve is very flat, this is suspicious.

**Table 3:** Seven plant species that showed inhibitory effect in sheep blood were subsequently tested in human blood. The agonists are ADP (5.0 *μ*M) and collagen (2.0 *μ*g/mL) and the obtained aggregations are shown in percent. Extracts are tested in 0.1 mg/mL end concentrations. The aggregation is shown for both extract and vehicle control. If the extract aggregation is 20% lower than that of the vehicle control inhibition is observed.

Plant	Extract	Agonist	% Aggregation (extract)	% Aggregation (vehicle control)	% Inhibition	Inhibition
*Amomyrtus luma* (leaf)	DCM : MeOH 1 : 1	ADP	57	73	22	Yes
		Collagen	62	87	29	Yes
	MeOH	ADP	66	70	6	No
		Collagen	68	70	3	No
	H_2_O	ADP	66	69	4	No
		Collagen	60	76	21	Yes

*Anthoxanthum utriculatum *(leaf)	MeOH	ADP	63	64	2	No
		Collagen	63	68	7	No

*Blechnum chilense* (leaf)	DCM : MeOH 1 : 1	ADP	73	77	5	No
		Collagen	74	73	−1	No
	MeOH	ADP	60	83	28	Yes
		Collagen	61	86	29	Yes

*Cestrum parqui* (leaf)	DCM : MeOH 1 : 1	ADP	61	84	27	Yes
		Collagen	67	84	20	Yes
	MeOH	ADP	70	70	0	No
		Collagen	71	71	0	No
	H_2_O	ADP	74	71	−4	No
		Collagen	64	68	6	No

* Lomatia ferruginea* (leaf)	DCM : MeOH 1 : 1	ADP	72	72	0	No
		Collagen	73	71	−3	No

*Luma apiculata* (leaf)	MeOH	ADP	70	67	−4	No
		Collagen	65	71	8	No
	H_2_O	ADP	46	84	45	Yes
		Collagen	54	85	36	Yes

*Pseudopanax laetevirens* (leaf)	DCM : MeOH 1 : 1	ADP	69	72	4	No
		Collagen	66	72	8	No
	MeOH	ADP	69	72	4	No
		Collagen	69	76	9	No

**Table 4:** Flow cytometry results from *L. apiculata* H_2_O extract, and *A. luma* DCM : MeOH 1 : 1 extract. Inhibitions of the platelet activation markers, PAC1 and CD62P are shown in percent compared with vehicle control. The agonist is ADP in 0.5 *μ*M and 20.0 *μ*M, and TRAP in 1.0 *μ*M and 5.0 *μ*M. Extracts are tested in 0.1 mg/mL end concentrations.

Plant extract	% Inhibition of PAC1 MFI				% inhibition of CD62P MFI			
	ADP 0.5 *μ*M	ADP 20.0 *μ*M	TRAP 1.0 *μ*M	TRAP 5.0 *μ*M	ADP 0.5 *μ*M	ADP 20.0 *μ*M	TRAP 1.0 *μ*M	TRAP 5.0 *μ*M
*Luma apiculata *(H_2_O extract)	34.4	20.1	74.1	8.0	38.9	28.1	80.7	3.5
*Amomyrtus luma *(DCM : MeOH 1 : 1 extract)	30.1	17.8	78.1	12.4	37.7	27.4	83.2	6.4
